# Spatial ecology of the invasive Asian common toad in Madagascar and its implications for invasion dynamics

**DOI:** 10.1038/s41598-023-29467-2

**Published:** 2023-03-02

**Authors:** Fulvio Licata, Gentile Francesco Ficetola, Mattia Falaschi, Benjamin J. Muller, Franco Andreone, Rodino Fetrarijahona Harison, Karen Freeman, Antonio T. Monteiro, Sophia Rosa, Angelica Crottini

**Affiliations:** 1 Centro de Investigação em Biodiversidade e Recursos Genéticos, CIBIO, InBIO Laboratório Associado, Universidade do Porto, Campus de Vairão, 4485-661 Vairão, Portugal; 2 Departamento de Biologia, Faculdade de Ciências, Universidade do Porto, 4099-002 Porto, Portugal; 3 BIOPOLIS Program in Genomics, Biodiversity and Land Planning, CIBIO, Campus de Vairão, 4485-661 Vairão, Portugal; 4 Department of Environmental Science and Policy, Università Degli Studi di Milano, 20133 Milano, Italy; 5 Laboratoire d’Écologie Alpine (LECA), Univ. Grenoble Alpes, CNRS, 38000 Grenoble, France; 6 Madagascar Fauna and Flora Group, BP 442, 501 Toamasina, Madagascar; 7 Museo Regionale di Scienze Naturali, Via G. Giolitti, 36, 10123 Torino, Italy; 8 ISSEDD (Institut Supérieur de Science, Environnement et Développement Durable), Université de Toamasina, Toamasina, Madagascar; 9 Centro de Estudos Geográficos (CEG), Instituto de Geografia e Ordenamento do Território (IGOT), Laboratório Associado TERRA, Universidade de Lisboa, Rua Edmée Marques, 1600-276 Lisboa, Portugal; 10 Istituto di Geoscienze e Georisorse, Consiglio Nazionale Delle Ricerche (CNR), Via Moruzzi 2, 56124 Pisa, Italy

**Keywords:** Ecology, Animal behaviour

## Abstract

Invasion dynamics are determined, among other aspects, by the spatial behaviour of invasive populations. The invasive toad *Duttaphrynus melanostictus* is spreading inland from the eastern coast of Madagascar, causing considerable ecological impacts. Understanding the basic factors determining the spread dynamics can inform management strategies and provide insights into spatial evolutionary processes. We radio-tracked 91 adult toads in three localities along the invasion gradient to determine whether spatial sorting of dispersive phenotypes is occurring, and investigate intrinsic and extrinsic determinants of spatial behaviour. Overall, toads in our study appeared as habitat generalists, and their sheltering behaviour was tied to water proximity, with toads changing shelter more frequently closer to waterbodies. Toads showed low displacement rates (mean = 4.12 m/day) and quite a philopatric behaviour but were able to perform daily movements of over 50 m. We did not detect any spatial sorting of dispersal-relevant traits nor sex- or size-biased dispersal. Our results suggest that toads are more likely to expand their range during the wet season, and that the range expansion is probably dominated by short-distance dispersal at this stage of the invasion, although a future increase in invasion speed is expected, due to the capacity for long-distance movements of this species.

## Introduction

The spread dynamics of invasive alien species (hereafter: invasion dynamics) represent the complex outcome of the interaction between population dynamics, biotic and abiotic features of the recipient environments^1^. Dispersal (i.e. the unidirectional movements of individuals towards or between breeding sites^2^) plays a prominent role in defining invasion dynamics, and can determine clearly distinct patterns. For instance, invasive populations often exhibit exponential range expansion following a process of niche filling, but in dispersal-limited species, this pattern may turn linear with a constant rate of spread^1^. Conversely, a stratified dispersal of the invasive population (i.e. when both short- and long-distance dispersers contribute to the range expansion^3^) may lead to an initial slow linear expansion, followed by a boosting phase determined by the coalescence of satellite colonies established beyond the range border by long-distance dispersers^1^. A similar accelerating pattern can be driven by spatial sorting of dispersive phenotypes at the invasion front, where dispersal-relevant traits are positively selected increasing dispersal capabilities and accelerating the spread rate of the invasion through generations^1,4^. Identifying the mechanisms underlying the spread of invasive alien species is key to forecasting and managing biological invasions but implies understanding the dispersal dynamics of the target species. This requires specific information about ongoing processes in invaded ranges, as dispersal is strongly context-dependent^5^.

The Asian common toad *Duttaphrynus melanostictus* is considered a highly problematic invasive species^6^. The recent introduction of this invasive toad in the seaport town of Toamasina in Eastern Madagascar—a global hotspot of biodiversity^7^—is particularly worrying due to its toxicity and the lack of resistance to toad toxins among native vertebrate predators^8^. At least one snake species is already known to suffer significant negative impacts from toad poisoning^9^. Invasive toads have spread over a large area (> 500 km^2^)^10^ and are deemed to be ineradicable with current management methods^11^. Understanding the invasion dynamics of this species and identifying the major factors driving its dispersal is pivotal to predicting the areas suffering the highest invasion risk in the coming years, and to plan management actions aimed at limiting its spread and mitigating its impacts. Amphibian bufonids (toads) exhibit the longest dispersal distances among anurans, however, these may substantially differ between and within species^5,12^. An outstanding example is the invasive cane toad (*Rhinella marina*) in Australia, where populations at the invasion front exhibit a fivefold higher rate of dispersal due to spatial sorting of dispersal-relevant traits^13^.

Both theoretical and empirical studies show that in many cases spatial sorting can emerge at a faster rate in behavioural traits rather than in external morphology^14–16^. The invasive Asian common toad in Madagascar does not show spatial sorting of morphological traits^17^, and investigating its spatial behaviour offers the opportunity to explore the evolutionary dynamics underlying the onset of spatial sorting in the early stages of a biological invasion. However, the straightforward identification of spatial sorting of behavioural traits may be hindered by a multitude of intrinsic and extrinsic factors which regulate toads’ spatial ecology. For instance, thermal and moisture conditions can strongly influence daily movement rates and spatial ethology of toads^18,19^. Toads breeding phenology—which is dictated by rainfall regime and water availability^20^—may also strongly influence their dispersal. Furthermore, toads’ polygynous mating system, with female choice determining the mating success^21^, might result in both male-biased dispersal or, if males show high territoriality, female-biased dispersal^5^. Sex-biased dispersal in invasive species can both facilitate or limit range expansion by changing the sex ratio and affecting the mate finding mechanisms at the invasion front^22^. Similar unbalancing effects on invasion dynamics may be driven by ontogenetic differences in spatial behaviour. Both sex- and size-biased dispersal may thus have important management implications.

Identifying the determinants of spatial behaviour in the invasive toad in Madagascar will allow us to disentangle the effects of spatial sorting from natural ecological processes, while providing important baseline data to inform management strategies. Toad dispersal can be quantified by measuring the distance travelled between shelter sites and the frequency of shelter-site change^13^. As shelter-site selection also represents toads’ behavioural response to environmental conditions^23^, exploring the sheltering behaviour can acquit the twofold purpose of quantifying dispersal rates and identifying dispersal limitations which shape the invasion dynamics of this species in Madagascar.

In this study, we used radio-tracking to assess the spatial ecology of invasive Asian toads in Madagascar to (1) test the hypothesis of spatial sorting of dispersal-related behavioural traits along the invasion gradient, and (2) identify intrinsic and extrinsic determinants of toad spatial behaviour, with the aim of obtaining insights into its invasion dynamics.

## Materials and methods

### The Asian toad in Madagascar

*Duttaphrynus melanostictus* is a species complex of medium-sized toads (body size in Madagascar, mean ± SD; females = 81.1 ± 11.4 mm; males = 72.5 ± 6.8 mm)^17^ native to South and South-East Asia^24^, with several invasive populations established in multiple regions of the world^25,26^. The Asian common toad normally breeds in lentic or slow-moving waters, both temporary and permanent, where females can lay up to 10,000 eggs (FL, unpubl. data). The breeding season is largely dependent on the rainfall regime^27^, and in Central Vietnam, where the climatic conditions are similar to the eastern coast of Madagascar^28^, takes place in the warmest months with monthly precipitation > 100 mm, and lasts until the beginning of the main rainy season, when the temperatures start to drop^29^.

The invasive population in Toamasina was likely introduced via unintentional transport in commercial containers between 2007 and 2011^30,31^, and the putative point of introduction was identified in the south-west of Toamasina^31^.

**Figure 1:**
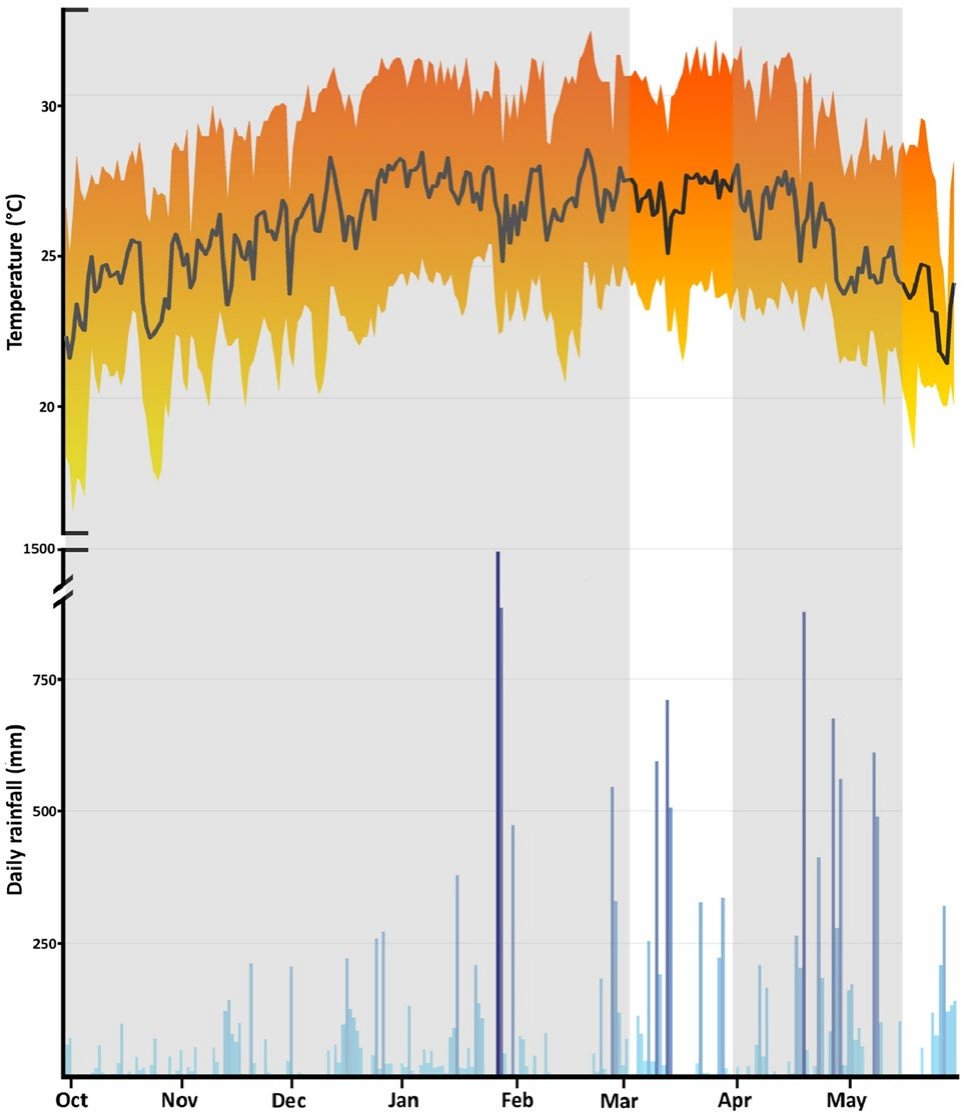
Average daily temperatures and rainfall during the study period (October 2018–May 2019). The grey areas indicate the periods of radio-tracking of the Asian common toad population in Toamasina (Madagascar). Hourly weather data were obtained from the local meteorological station located at Toamasina airport.

The invasion is estimated to spread at a rate of approximately 2 km year^−1^^32^, expanding in all landward directions and habitats^9,10^, which include the urban areas of Toamasina, and the surrounding countryside characterised by croplands, mixed shrubland, and some remnant patches of secondary humid lowland forest^33^. This region is characterised by a tropical rainforest climate^34^ with a drier season (August–November; range average rainfall = 85–124 mm/month) and a wet season (December–July; range average rainfall = 181–343 mm/month), with average monthly temperatures ranging from 21 °C (July) to 27 °C (February)^35^. This climate is apparently highly suitable for the *D. melanostictus* lineage that is spreading in Madagascar^28^. Our study period was characterised by a slightly longer drier season than usual, with the first heavy rainfalls in January (Fig. 1).

### Study area

**Figure 2:**
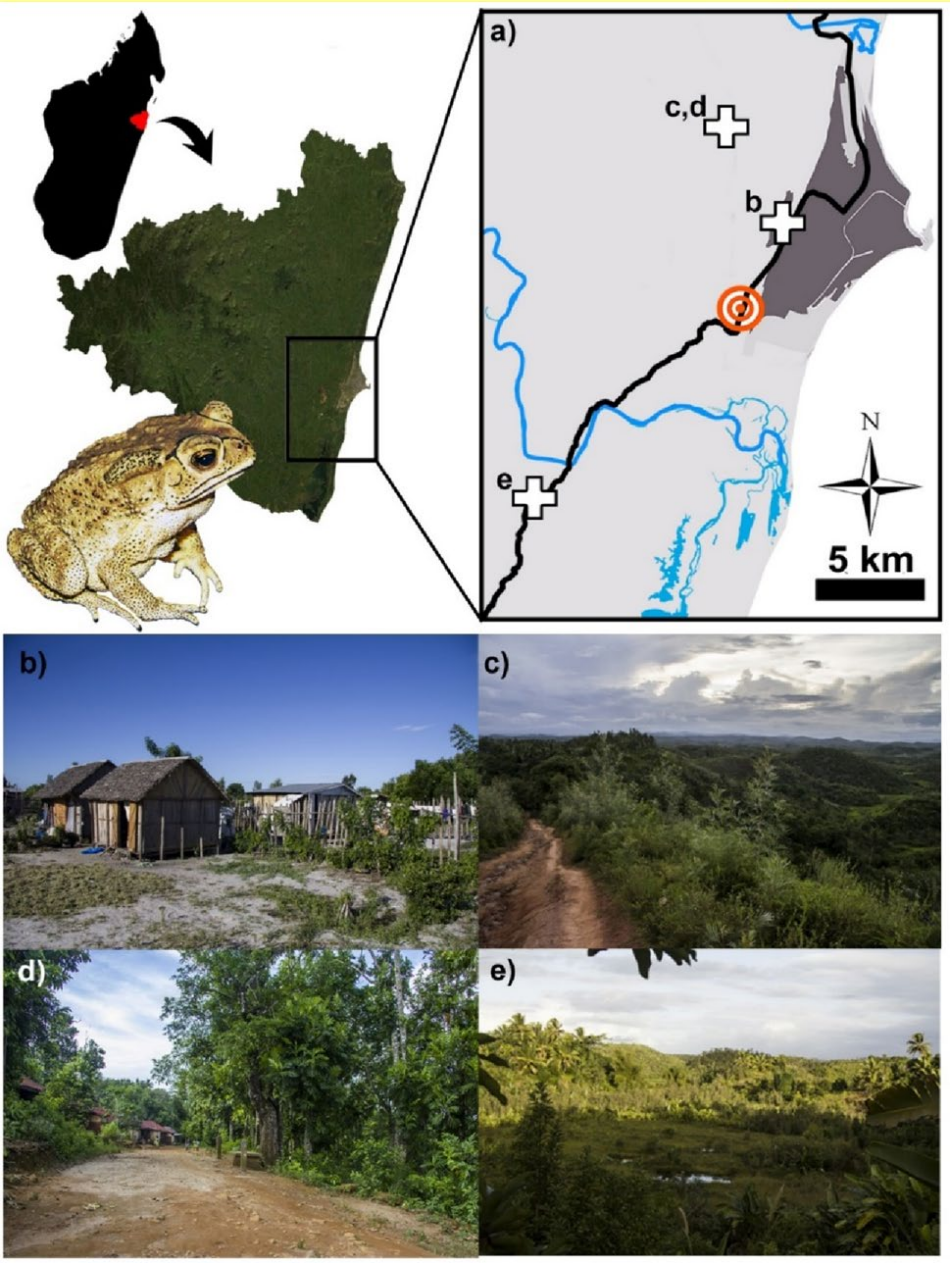
Map of the study area with the sites (white crosses) where the radio-tracking study of the Asian common toad was conducted (**a**) showing the putative point of introduction^31^ (red bullseye), and the main road connecting Toamasina to Antananarivo (black line). Representative photographs of the main landscapes of the study sites: (**b**) Ambodisaina; (**c,d**) Vohitrambato route; (**e**) Invasion forefront. We created the map in Fig. 2a by using the software ArcGIS (vers. 10.2; https://www.arcgis.com/index.html).

We captured adult toads from three different localities (Fig. 2a) to study behavioural variation between habitats, and between the core and the front of the invasion, as follows:

1. *Ambodisaina* (18.148°S, 49.363°E) is a suburban quarter of Toamasina located 5.4 km from the estimated introduction point, where the toads established around 2014. This site is flat, near a natural wetland extensively used for paddy fields, and includes several artificial pools used for fish culture and private use (Fig. 2b).
2. *Vohitrambato Route* (from 18.139°S, 49.346°E to 18.103°S, 49.322°E), located 5.6–9.8 km from the estimated introduction point. It is a route which traverses two small villages (< 50 houses) in the countryside, interspersed into a landscape characterised by a hilly topography (max. elevation 80 m), rich in wet areas, paddy fields and streams, and dominated by degraded forest and mixed shrubland, with a remnant patch of secondary rainforest^9^. Toads likely arrived here between 2014 and 2017^32^(Fig. 2c,d).
3. *Invasion forefront* (18.273°S, 49.254°E), 13 km south of the estimated introduction point, where the invasion front was identified in 2019^17^. Toads were found by surveying nearby three small settlements located less than 1 km apart, along the major road which connects Toamasina to the capital Antananarivo (“Route Nationale 2”), in a landscape dominated by grassland, mixed shrubland, and paddy fields (Fig. 2e).

### Radio-tracking methodology

We conducted radio-tracking over 212 days, between the end of the drier season (23 October 2018) and the end of the rainy season (23 May 2019), excluding the period from 4 to 31 March 2019 (Fig. 1). After capture, SVL (snout-vent length; mm) and mass (g) of individuals were measured. Sex determination was based on the external characters, with females larger and heavier than males, which in turn have thicker forearms, nuptial pads and develop an orange gular region^30,36^. Each toad was fitted with a flexible belt around the waist made of silicone rubber capillary tubing (ca. 0.05 g)^37^, holding a radio-transmitter (mass = 2.5 g; model NTF-6-1; Lotek Systems, Ontario, Canada). Toads were released back at their exact capture site within 24 h, and we discarded the first movement from the spatial ecology analysis since it included the observation of active toads.

We located toads in their shelters between 11:00 h (approx. solar noon) and 21:00 h (~ 3 h after sunset). After a toad was located, we recorded the GPS coordinates (Garmin GPSMAP60 CSx; Garmin, Kansas, USA), time of the observation, and shelter type. We used GPS locations to calculate the distance between consecutive shelter sites. Although breeding activity could have contributed to the spatial behaviour of tracked toads, only movements associated with the observation of tracked toads breeding were considered reproductive movements.

To investigate sheltering behaviour, we determined the type of shelter, distinguishing between natural or anthropogenic, and whether toads changed shelter between consecutive observations. We measured the net displacement as the Euclidean distance between the start and the end point of the tracked toad, and the total displacement as the sum of all movements, while we obtained the path straightness by dividing the net displacement by the total displacement.

### Environmental variables

As weather variables, we considered average temperature (°C), average relative humidity (%), and average precipitation rates (mm) during wet hours (i.e. only considering the rainy hours) between consecutive toad observations. Hourly weather data were obtained from the meteorological station located at Toamasina airport (5–22 km from the study sites).

As topographical variables, we considered the slope (º) and aspect. Since aspect is a circular variable, the cosine and sine transformations were applied to obtain a continuous variable stressing the north–south and east–west gradients, respectively (northness; − 1 south-exposed to 1 north-exposed; eastness: − 1 west-exposed to 1 east-exposed)^38^. Topographical variables were derived from ASTER Global Digital Elevation Map (30 m spatial resolution)^39^.

As habitat variables, we considered four land cover classes (forest, shrubland, grassland, and barren land). Furthermore, as toads use water bodies not only for breeding purposes, but also to hydrate^18^, we also measured the distance of shelters to the nearest permanent water body (i.e. natural and artificial ponds/rice fields), which was estimated using a Euclidean function. Lastly, we considered the density of roads, as they can be used as dispersal corridors by toads^40^. Road density was based on a road map obtained from the Geofabrik OpenStreetMap server (www.geofabrik.de). To obtain land cover, a supervised classification of Sentinel-2 Copernicus satellite imagery was performed using the machine learning approach Random Forests^41^ (see Appendix S1, Table S1).

Before analyses, all data were downscaled to obtain 20 m spatial resolution grid cells, which maintained enhanced habitat characterisation while reducing the computational demand for the modelling procedure. Raster files were manipulated using the R package *raster*^42^.

### Statistical analyses

Body condition can influence movement performance of toads^43^. Therefore, we calculated the Residual Index (RI), which is considered a reliable body condition index^44^ and is obtained by extracting the residuals of the regression of mass against SVL (both ln-transformed), for each toad. The frequency distribution of toad movements (i.e. dispersal curve) was fitted with exponential (ln-transformed frequency *vs.* distance) and power-law curves (ln-transformed frequency *vs.* ln-transformed distance).

We used linear mixed-effect models (LMMs) to assess the factors related to toad movements (i.e. distance between shelters), excluding migratory movements. For this analysis, we included sex, body size (SVL) and body condition (RI) of toads as potential predictors of distance travelled. To assess the relationship between distances travelled by toads and weather variables, we included the average humidity and temperature between observations, and the rainfall in only the wet hours between observations. We also included the topographical variables (i.e. slope and aspect) at the starting point of the movement, to assess for geotactic responses in the spatial behaviour of toads. The distance from waterbodies may be a potential driver of movement in toads^45^, therefore, we included the distance from the nearest waterbody at the starting point of the movement among the habitat variables to be tested. As distances travelled may increase through time, we also included the number of hours between consecutive observations (ln-transformed). Methodological standards recommend using radiotelemetric equipment lighter than 10% of the body mass of the animal^46^, although there is little evidence that exceeding this threshold negatively impacts amphibians’ behaviour^47^. The relative radio-transmitter mass widely varied among the toads tracked in this study (range = 2.8–12.7%; mean ± SD = 7.2 ± 2.4%). To assess whether the radio-tracking equipment could affect toad movements, we also included the relative transmitter mass among the independent variables of our models. Finally, we included the locality to assess differences between populations, while toad ID was included as a random effect to account for inter-individual variation. Prior to building the models, we ln-transformed the response variable to reduce skewness.

To further assess the role of water proximity on sheltering behaviour, we calculated for each toad the mean distance of shelters from waterbodies, which was ln-transformed and used as a response variable in a linear regression. For this analysis, we used sex, SVL, RI of toads, and the average value of each weather variable during the tracking period as explanatory variables.

We used binomial generalised linear mixed-effect models (GLMMs) to test whether the probability that a toad changed shelter was related to weather and topographic variables, toads’ sex, SVL, and RI. As further explanatory variables we included the distance from waterbodies of the shelter previously used, and the time between observations (ln-transformed), as well as the relative radio-transmitter mass, while toad ID was included as a random effect.

We tested the hypotheses of spatial sorting, sex- and size-biased dispersal on movement patterns of toads, by using path straightness (logit-transformed), net and total displacement (ln-transformed) as response variables. The accuracy of these variables may be strongly affected by inter-individual variation of radio-tracking data (e.g. length of tracking period, number of observations per toad). Therefore, we performed weighted least squares regressions, excluding those individuals with fewer than three observations (i.e. the minimum number of locations needed to estimate path straightness). Weights were given by using the number of observations of toads. As independent variables, we considered the distance from the introduction point, toads’ sex, SVL and RI, and the average weather conditions (i.e. temperature, humidity, and rain during wet hours) during the tracking period. We also included the length of the tracking period (ln-transformed), as it can be strongly related to the distance travelled by toads.

To assess the effect of habitat on shelter-site selection, we used a grid-based approach which allowed us to account for habitat availability at the individual level. For this analysis, we only selected consecutive daily observations to exclude the movements not accounted for by the tracking regime. We created a grid whose centre was the cell at which the toad was located before performing the movement (i.e. starting cell), with all grid cells being potentially selected the following day by the toad (i.e. arrival cells). To reduce the computational demand for the modelling procedure, we limited the size of the grid to 49 cells spaced 20 m apart, therefore we excluded all movements above 65 m (approx. 1.5% of movements), which fell outside the range of the grid. Preliminary analysis using GLMMs showed that toad ID used as a random factor did not explain any variation of the dependent variable (i.e. presence/absence of the toad in a specific cell). Therefore, for this analysis, we excluded toad ID as a random effect and used generalised linear models (GLMs) with binomial error distribution to test two different hypotheses that could explain the probability that a toad moved into a cell. First hypothesis (H1): a cell is selected for its habitat characteristics. To test H1, we used land cover values of the arrival cells as explanatory variables (i.e. the proportion of barren land, grassland, scrubland, forest; density of roads and distance from the nearest waterbody). Second hypothesis (H2): a cell is selected for its habitat dissimilarities with respect to the starting cell. To test H2, we used the difference of values between the arrival cells and the starting cell for each land cover variable (e.g. proportion of barren land in the arrival cell minus proportion of barren land in the starting cell). Since the probability of a cell being selected decreased with the distance from the starting point, we included distance of the cell from the starting point and its quadratic term in both models. The correlations between explanatory variables were always < 0.7, indicating limited collinearity^48^. Prior to running the models, all continuous independent variables were scaled (mean = 0; SD = 1) to allow comparison of estimated parameters.

The modelling procedure was the same for all models: models were built using all combinations of explanatory variables without their interactions, and ranked on the basis of the Akaike’s Information Criterion (AIC)^49^. AIC corrected for small sample sizes (AIC_c_) was used to rank the weighted least square regressions of path straightness, net, and total displacement, since we had only one value per toad. To select the set of candidate models, we removed all models which had a simpler nested model with lower AIC (or AIC_c_)^50^. For each model, we calculated the AIC (or AIC_c_) weight, which indicates the relative likelihood of a model given the data and the candidate set of models. For models with binomial error distribution, test statistics were calculated using the likelihood-ratio test. Models were run in R environment^51^ using the packages *lme4* for mixed effect models^52^, *lmerTest* to obtain test statistics^53^ and *MuMIn* for model selection^54^.

## Results

### Tracked toads

We tracked 44 toads in the suburban quarter of Ambodisaina (24 males and 20 females), 44 toads along the Vohitrambato Route (14 males and 30 females), and three toads at the invasion forefront (1 male and 2 females), where adults were found in low numbers (Supplementary Table S2). Four toads from Vohitrambato Route did not complete the tracking period either because they died (n = 1), lost the harness (n = 2) or could not be located (n = 1). The tracking period was on average 4.9 ± 2.9 (SD) days, with locations obtained on average every 52.1 ± 30.6 h (Supplementary Table S2). Toads from the different localities did not differ significantly in body size (SVL; ANOVA: F_2,88_ = 0.94, *P* = 0.3), while a significant effect was found for body condition (F_2,88_ = 3.31, *P* = 0.04), with toads from Vohitrambato Route being slightly leaner than those from Ambodisaina (Tukey’s post-hoc test; *P* = 0.03).

### Toad movements

**Figure 3:**
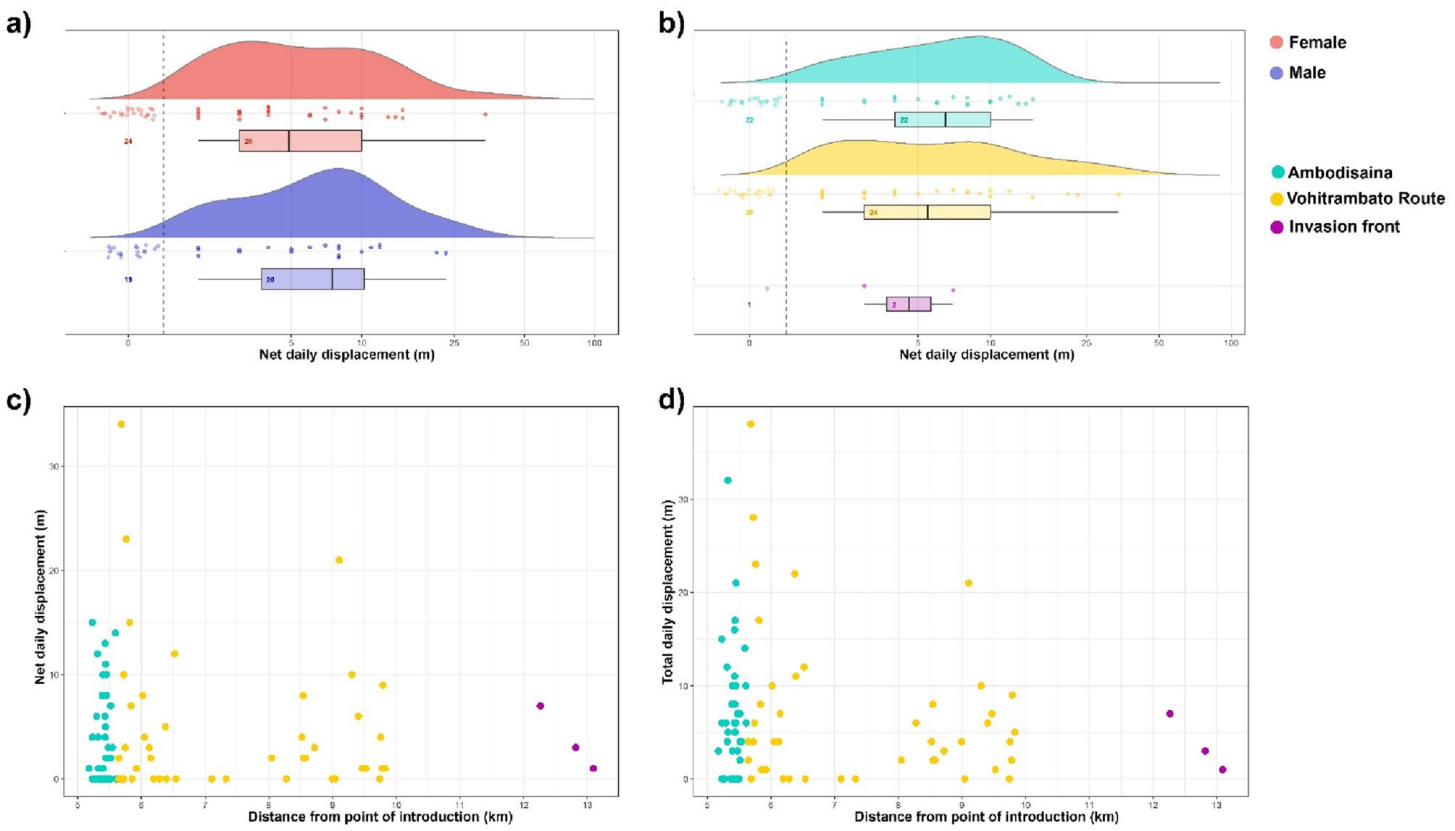
(**a**) Frequency distribution of daily movements of 72 Asian common toads (the individuals for which daily movements were available) in Toamasina (Madagascar), divided by sex (females = red, males = blue) and (**b**) locality (Ambodisaina = sky blue, Vohitrambato Route = yellow, Invasion front = purple); (**c**) scatterplot of net daily displacement of tracked toads in relation to the distance from the introduction point; (**d**) scatterplot of total daily displacement of tracked toads in relation to the distance from the introduction point, Colors in panel (**c**) and (**d**) indicate the different localities, such as in panel (**b**). Box and density plots are plotted excluding non-movements. The number of daily movements used to create the boxplot is indicated within the boxplot. R code used to obtain this plot was modified from Hodges et al.^55^.

The mean distance travelled between shelters by toads within a single night was 15.3 ± 14.6 m (Fig. 3), with maximum long-distance daily movements of 68.9 m (Supplementary Table S2). Toads showed a right-skewed, leptokurtic dispersal curve (skewness = 2.2; kurtosis = 8.1), which was best fitted by an inverse power relationship (adjusted R^2^ = 0.62). Dispersal curves were similar for both sexes (males: skewness = 2.3, kurtosis = 8.9; females, skewness = 2.1, kurtosis = 7.2). During the tracking period, a male moved 114 m to reach a rice paddy, where it stayed until the end of the tracking period. We considered this as a reproductive, migratory movement, and excluded it from subsequent analyses.

According to the best-AIC model, the distances between consecutive shelters increased with increasing humidity (LMM; *B* ± SE = 0.25 ± 0.9; *F*_1,210.7_ = 6.62, *P* = 0.01), and with the time between observations (*B* ± SE = 0.36 ± 0.09; *F*_1,219.3_ = 15.01, *P* < 0.001). No different predictors were included in competing models (Supplementary Table S3).

Total displacement by the end of the tracking period was on average 27.9 ± 31.1 m, with an average daily displacement of 6.3 ± 7.4 m (Supplementary Table S2). The best model explaining toads’ total displacement showed a positive relationship with the length of the tracking period (weighed regression; *B* ± SE = 0.52 ± 0.16; *F*_1,89_ = 10.54, *P* = 0.001) (Supplementary Table S4).

The average net displacement was 16.5 ± 19 m over the tracking period, which translates into an average net daily and yearly displacement of 4.1 ± 5.8 m and 1506.8 ± 2121.8 m, respectively (Supplementary Table S2). No predictors explained variation of toads’ net displacement and path straightness (*P* always > 0.05), with all competing models showing only a slightly better fit than the null model (Supplementary Table S4).

### Sheltering behavior

**Figure 4:**
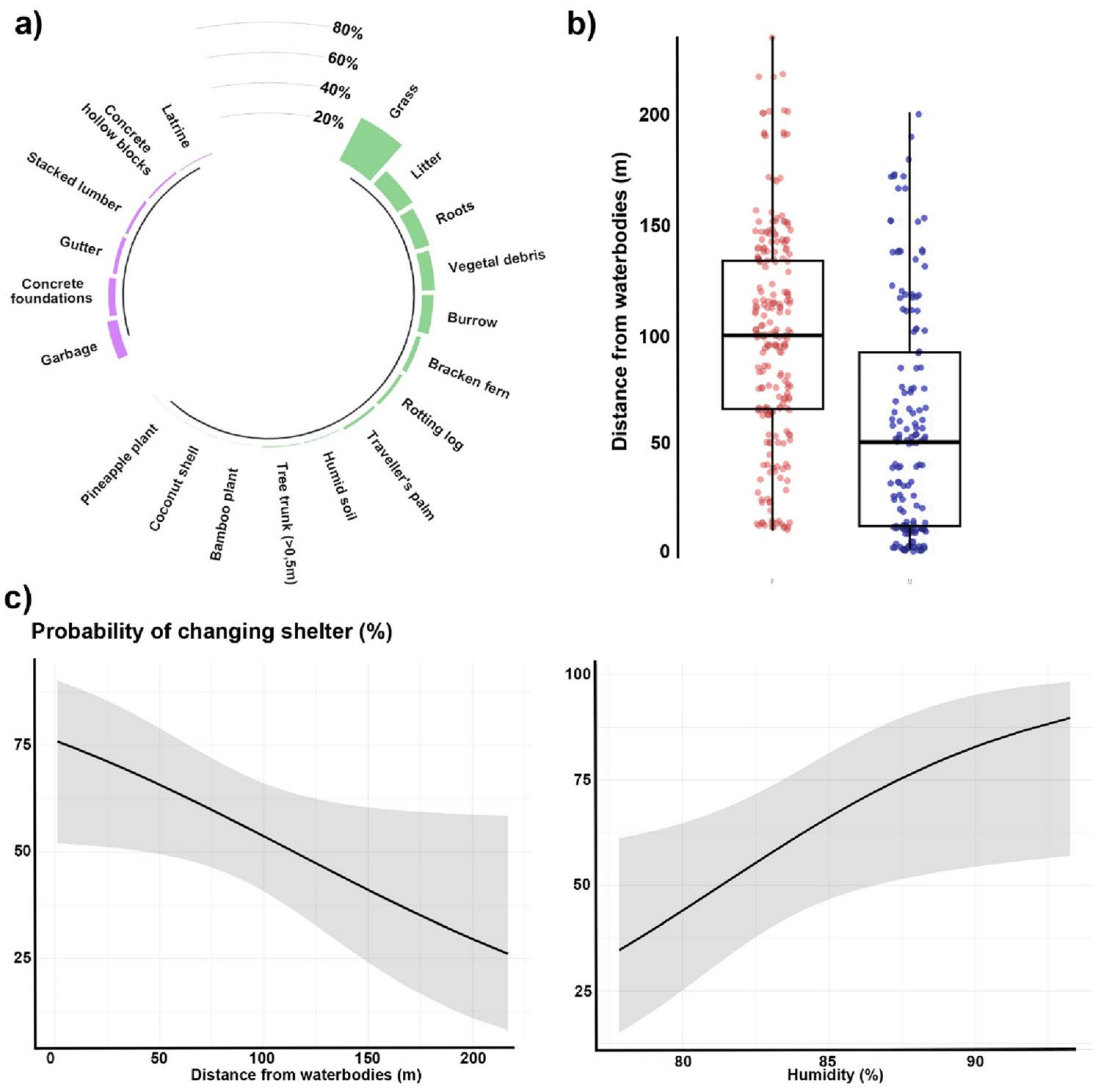
Sheltering behaviour of the Asian common toad in Toamasina (Madagascar). (**a**) Type of shelters (in green natural shelters, in purple anthropogenic shelters); (**b**) boxplot of distances of shelters from the nearest water bodies (in blue—right—males, in red—left—females); (**c**) predicted probability of changing shelter in relation to the distance from the nearest permanent water body (left) and in relation to humidity (right).

Toads changed shelter in 55.1% of the observations obtained on successive days, using both natural and anthropogenic shelters, and principally hiding in grass (31.5% of the observations), litter (12%), and beneath tree roots (10.2%) (Fig. 4a). Toads sought shelter up to 235 m away from waterbodies, with the best model explaining shelter distance from waterbodies including sex as the only significant predictor, with males seeking shelter closer to water than females (linear regression; *B* ± SE = -0.99 ± 0.22; *F*_1,88_ = 18.6, *P* < 0.001; Fig. 4b, Supplementary Table S2).

The probability that toads change shelter significantly increased with increasing time between observations (binomial GLMM; χ^2^ = 6.10, df = 1, *P* = 0.01) and humidity (χ^2^ = 5.39, df = 1, *P* = 0.02; Fig. 4c), while it significantly decreased moving away from waterbodies (χ^2^ = 5.48, df = 1, *P* = 0.01; Fig. 4c). The best model explaining the probability that toads change shelter also included as a predictor the sex of individuals, which, however, had a non-significant effect (*P* > 0.05) (Supplementary Table S3). Conversely, toads’ sex was not included in a second, nested competing model, which also showed a nonsignificant effect of the distance from waterbodies (χ^2^ = 3.36, df = 1, *P* = 0.06).

Toads inhabited all types of habitats available. The only variable explaining the probability that a toad moved into a cell based on its habitat characteristics was the quadratic distance of the arrival cell (χ^2^ = 762.7, df = 2, *P* < 0.001). Conversely, when considering the habitat dissimilarities between starting and arrival cell, the best model included the difference in barren land between starting and arrival cells, which, however, was not significant (χ^2^ = 3.05, df = 2, *P* = 0.08) (Supplementary Table S5).

## Discussion

Tracked toads were habitat generalists, showing low displacement rates and philopatric behaviour, but proved able to perform long-distance movements. Dispersal-relevant ethological traits did not change along the invasion gradient, although the low number of tracked toads at the invasion front did not allow us to firmly exclude a spatial sorting effect. Movements and sheltering behaviour were mostly related to moist conditions and water proximity. We found differences in the spatial behaviour between sexes, with males living closer to water bodies than females.

Altogether, these results support empirical evidence that the range expansion is probably dominated by short-distance leading edge dispersal at this stage of the invasion, however, the long-distance dispersal potential of the tracked toads suggests the possibility of a future increase in invasion speed.

### Effects of weather and habitat on spatial behaviour

Toads were more active with humid and wetter weather conditions; therefore, the wet season is expected to be the most favourable period for toad dispersal. Moisture conditions are correlated with evaporative loss rates and are a well-known driver of movement in toads^56^ and amphibians in general^57^. However, weather conditions had a weak effect on the spatial behaviour of toads (Supplementary Table S3), suggesting that further unaccounted factors, such as food availability, breeding phenology, and reproductive status, may play an important role, as well as the method used (i.e. locating toads maximum once per day), which likely underestimated the distances travelled overnight.

Toad movements did not differ among localities, indicating similar spatial strategies despite the environmental context. This is also reflected in the habitat preferences, with toads found in all types of habitats.

### Factors determining differences between individuals

Spatial ecology can also be influenced by intrinsic individual factors^58^. We found that body condition and body size did not affect movements and sheltering behaviour. Body size and body conditions can strongly correlate with dispersal in amphibians, and may have a direct effect on behavioural and physiological locomotor processes^5^. In this study, the tracked individuals showed similar philopatric behaviour, which could explain the lack of size-dependent variation in the spatial behaviour of adult toads. However, the coarse and short-term tracking regime in this study could also have hindered the detection of the effect of body size and body condition on toad movements.

Our results show that both sexes had similar spatial ecology, although they differed in their spatial distribution. Adult males preferred waterside habitats, as documented in other anurans^21,59^. Asian toads are considered prolonged breeders^27^, and males can exhibit a fluctuating continuous reproductive activity^60,61^, which might induce individuals to stay closer to the breeding sites to maximise the mating probabilities.

Path straightness is a focal metric of spatial ecology often used to determine the dispersiveness of a species and to pinpoint potential trends of adaptive dispersal behaviour, as it represents heritable traits that can undergo rapid evolutionary selection^62,63^. Contrary to what has been documented in the invasive cane toad in Australia^62,64^, we did not detect spatial sorting in path straightness or other spatial ecology metrics considered (i.e. net and total displacement; Fig. 3c,d). This could be due to multiple, non-exclusive reasons, including the short invasion history of this species in Madagascar^17^ or the absence of trait shifts at the expanding population edges^4^. For instance, imperfect dispersal heritability might cause a weak response to spatial selection^63^ and demographic stochasticity might overwhelm the spatial sorting effect^65^. Similarly, limited breeding dispersal (e.g. high site fidelity) can also hinder spatial sorting of dispersal-relevant traits, decelerating the range expansion. Lastly, large sample sizes can be required to detect any spatial sorting effect when behavioural changes are too small due to the short invasion history or there is high interindividual trait variation^4^. The lack of evidence of spatial sorting in our study could therefore also be due to the small sample size from the invasion front, where adult toads were found in very low densities.

Baseline data coupled with long-term monitoring of this invasive population will allow understanding whether invasive toads in Madagascar can undergo spatial sorting of dispersal-relevant traits, while at the same time will offer the opportunity to explore what interspecific differences in spatial behaviour may play a role in the spatial evolutionary dynamics in invasive species.

### Insights into the spreading dynamics of toads

Malagasy Asian common toads’ displacement rates (i.e. approx. 4 m per day and 1.5 km per year) stand among the lowest in bufonids^66,67^, and are largely inferior to most of the values documented in both native and invasive cane toad populations^13^.

Similar to our results, recent estimates indicate that the Asian common toad in Madagascar is spreading at an average rate of ca. 2 km per year following a constant linear spread rate^32^, which is a pattern often associated with limited species dispersal^1^. This likely explains why *D. melanostictus* is invading Madagascar at a much slower rate than the cane toad at the early stages of the invasion in Australia (10–15 km/year^68^). Asian toads are smaller than cane toads and have significantly shorter hind limbs, which may result in comparatively lower locomotor performances^69^. Furthermore, their comparatively small body mass might limit resistance to evaporative water loss^70^ and thermal tolerance^71^. Further extrinsic, context-dependent factors may have limiting effects on the spread of invasive toads in Madagascar. For instance, conspecific density and breeding success can be important drivers of dispersal, as well as the distance between breeding sites in the landscape^5^. Future studies focusing on these aspects are needed to elucidate the mechanism underlying the colonisation dynamics and rates of emigration or immigration in breeding sites.

The low net displacements of Asian common toads were due to philopatric movements rather than limited activity (toads changed shelter over 50% of times on consecutive nights; see results), and long-distance movements were infrequent (i.e. 2.5% of movements recorded during the dispersal-favourable season), suggesting that range expansion is probably dominated by short-distance leading edge dispersal at this stage of the invasion. However, leptokurtic distribution of toad movements was not exponentially bounded, indicating long-distance dispersal capabilities at the population-level, which, over time, is expected to increase the invasion speed^72^. Furthermore, we likely underestimated the long-distance dispersal potential of this species. In fact, our study did not include five toads that were lost at the beginning of the tracking period (i.e. they potentially moved offsite), therefore we could have missed their dispersive behaviour^73^. Additionally, if long-distance dispersive behaviour occurs infrequently at the individual level, our tracking period may not have been long enough to detect these movements. Despite the large number of tracked toads, we could not assess inter-individual variation in space use across seasons or define the home range size of this species, for which longer radiotelemetric studies to obtain a full understanding of the spatial ecology are needed. Lastly, as dispersal potential in amphibians can be age-dependent, with juveniles more dispersive than adults^5^, further studies on natal dispersal are warranted. In fact, as juvenile toads are found to outnumber adults at recently-invaded localities, natal dispersal could have an important role in the range expansion of this species^17^.

## Conclusion

We provided the first insights into the spatial ecology of one of the most problematic invasive amphibians^6^, thus our results have important implications for modelling and management applications, both in Madagascar and elsewhere.

For instance, to maximise the outcomes of hand-removal and trapping campaigns in Madagascar, management actions should be focused during the dry season (i.e. August–November), when toads are more sedentary^74^, and preferably not in the immediate vicinities of water bodies, to target females. Furthermore, since toads sheltered underneath piles of stacked lumber, vegetation debris, garbage, and hollow concrete blocks (Fig. 4a), these and similar goods should be prioritised for biosecurity controls, to avert accidental transport of toads to new areas. Our work stresses the importance of fundamental studies to predict invasion outcomes, especially looking through the prism of the invasion syndrome framework^75^. This approach aims to identify shared features among invasion events which may result in predictable invasion dynamics and impacts that can ultimately be addressed through similar management responses^75^. Due to the lack of native bufonids, the toad invasion in Madagascar has clear similarities with the cane toad invasion in Australia, thus the management framework of the toad invasion in Madagascar could benefit from building upon the intensively studied cane toad model system. For instance, tadpoles traps which exploit cannibalism in early life-stages^76^ or acoustic and light traps for adults could also be tested for the toad invasion in Madagascar^77^. Although further research is needed to exclude spatial sorting in the toad invasion in Madagascar, in this study we highlighted important spatial behavioural traits, which reflect on the invasion potential. The limited displacement rates, and the different spatial distribution of sexes have meaningful implications for the management of the Asian common toad in Madagascar, thus requiring fine-tuning of management strategies to fully exploit the tools available to cope with toad invasions^74^.

## Supplementary Information

Supplementary Information.

Supplementary Table S1.

Supplementary Table S2.

Supplementary Table S3.

## Data Availability

The datasets used in the current study are available in the supporting information (Supplementary Table S2, S6, S7).
