# Supplementary material for: Spatial ecology of the invasive Asian common toad in Madagascar and its implications for invasion dynamics
Source: Sci Rep. 2023 Mar 2;13:3526. doi: 10.1038/s41598-023-29467-2 (PMC9981762; doi:10.1038/s41598-023-29467-2)
Supplement: Supplementary file 1 — Supplementary Information. [file 41598_2023_29467_MOESM1_ESM.docx]

**Supplementary materials**

**Spatial ecossslogy of the invasive Asian common toad in Madagascar and its implications for invasion dynamics**

Fulvio Licata, Gentile Francesco Ficetola, Mattia Falaschi, Benjamin Muller, Franco Andreone, Rodino Fetrarijahona Harison, Karen Freeman, Antonio Monteiro, Sophia Rosa, Angelica Crottini

**List of supporting information:**

- **Appendix S1: Supervised classification of Sentinel-2 Copernicus satellite imagery**
- **Table S1: Confusion matrix for all land cover classes used in the analysis of the habitat preferences of the invasive Asian common toad in Madagascar.**
- **Table S3: List of models (LMs, LMMs, and GLMMs) explaining the sheltering behaviour of the Asian common toad in Toamasina (Madagascar).**
- **Table S4: List of best weighted least square regressions explaining the spatial ecology metrics of the Asian common toad in Toamasina (Madagascar).**
- **Table S5: List of binomial GLMs explaining the probability of habitat selection in the Asian common toad in Toamasina (Madagascar).**

**Appendix S1**

**Supervised classification of Sentinel-2 Copernicus satellite imagery**

The Sentinel-2 data captured the study area on 07/11/2017 and all the spectral bands at 10 m spatial resolution (Blue, Green, Red, Near-infrared bands) were considered for the classification. The classification considered four land cover classes (forest, shrubland, grassland, and barren land) and a set of 40 training sites.

Validation was performed though a confusion matrix that related reference data (forest, n = 28; shrubland, n = 43; grassland, n = 42; barren, n = 47; water, n = 38) to map data, to estimate overall producers’ and users’ accuracy. The overall accuracy statistics showed that the map is highly accurate (0.94). The users’ accuracy of all land cover classes was in the range 0.90 – 1.0, indicating the high reliability of the map (classes present in the map are actually on the ground). Producers’ accuracy values were in the range 0.84 – 1.0, demonstrating the good quality of the map and a slight tendency of the classification to underestimate the areas covered with grass (0.88) and scrub (0.84) (Table S4).

Table S1. Confusion matrix for all land cover classes used in the analysis of the habitat preferences of the invasive Asian common toad in Madagascar.

| **Map/Reference** | **water** | | **barren** | | **grass** | | **scrub** | | **forest** | | **Producer's accuracy** | |  |
| --- | --- | --- | --- | --- | --- | --- | --- | --- | --- | --- | --- | --- | --- |
| **water** | | 38 | | 0 | | 0 | | 0 | | 0 | | 1.000 | |
| **barren** | | 0 | | 47 | | 1 | | 0 | | 0 | | 1.000 | |
| **grass** | | 0 | | 0 | | 37 | | 4 | | 0 | | 0.881 | |
| **scrub** | | 0 | | 0 | | 4 | | 36 | | 0 | | 0.837 | |
| **forest** | | 0 | | 0 | | 0 | | 3 | | 28 | | 1.000 | |
| **User's accuracy** | | 1.000 | | 0.979 | | 0.902 | | 0.900 | | 0.903 | |  | |

Table S3 - List of models (LMs, LMMs, and GLMMs) explaining the sheltering behaviour of the Asian common toad in Toamasina (Madagascar). The table shows the number of parameters estimated in the model (K), AIC score of the model, AIC difference with the best model (ΔAIC), the relative weight of the model with respect to the candidate set of models (*w*AIC), variation explained by the fixed effects (R^2^_M_ = marginal R^2^) or by both fixed and random effects of the model (R^2^_C_ = conditional R^2^). Models which had a simpler nested model with lower AIC were removed. Only models with ΔAIC < 2 and null models are shown. In the best models, significant variables are highlighted in bold. Time btw obs = Time between observations (hours); Rain = precipitation rate during wet hours.

| **Response** | |  |  |  |  | **K** | **AIC** | **ΔAIC** | **wAIC** | **R^2^_M_** | **R^2^_C_** | **Adj R2** |
| --- | --- | --- | --- | --- | --- | --- | --- | --- | --- | --- | --- | --- |
|  | **Independent variables** | | |  |  |  |  |  |  |  |  |  |
| Distance between shelters | | |  |  |  |  |  |  |  |  |  |  |
|  | **Time btw obs** + **Humidity** | | |  |  | 5 | 802.78 | 0.00 | 0.69 | 0.10 | 0.31 | - |
|  | **Time btw obs** | |  |  |  | 4 | 804.45 | 1.67 | 0.30 | 0.07 | 0.26 | - |
|  | […] |  |  |  |  |  |  |  |  |  |  |  |
|  | *Null* |  |  |  |  | 3 | 816.32 | 13.55 | 0.00 | 0.00 | 0.23 | - |
|  |  |  |  |  |  |  |  |  |  |  |  |  |
| Distance of shelters from waterbodies | | | |  |  |  |  |  |  |  |  |  |
|  | **Sex** + Temperature | |  |  |  | 4 | 275.67 | 0.00 | 0.57 | - | - | 0.22 |
|  | **Sex** |  |  |  |  | 3 | 276.20 | 0.53 | 0.43 | - | - | 0.20 |
|  | […] |  |  |  |  |  |  |  |  |  |  |  |
|  | *Null* |  |  |  |  | 2 | 293.57 | 17.90 | 0.00 | - | - | 0 |
|  |  |  |  |  |  |  |  |  |  |  |  |  |
| Probability of changing shelter | | |  |  |  |  |  |  |  |  |  |  |
|  | **Time btw obs** + **Humidity** + **Distance water** + Sex | | | | | 6 | 278.11 | 0.00 | 0.32 | 0.14 | 0.38 | - |
|  | **Time btw obs** + **Humidity** + Distance water | | | | | 5 | 278.94 | 0.83 | 0.21 | 0.12 | 0.37 | - |
|  | […] |  |  |  |  |  |  |  |  |  |  |  |
|  | *Null* |  |  |  |  | 2 | 297.84 | 13.16 | 0.00 | 0.00 | 0.26 | - |
|  |  |  |  |  |  |  |  |  |  |  |  |  |

Table S4 - List of best weighted least square regressions explaining the spatial ecology metrics of the Asian common toad in Toamasina (Madagascar). Number of parameters estimated in the model (K), AIC_c_ score of the model, AIC_c_ difference with the best model (ΔAIC_c_), relative weight of the model with respect to the candidate set of models (*w*AIC_c_), variation explained by the models adjusted for the number of parameters (Adj R^2^). Complex models having simpler nested models with lower AIC_c_ values are excluded. Only models with ΔAIC < 2 and null models are shown. Significant variables are highlighted in bold.

| **Response** | |  |  |  |  | **K** | **AIC_c_** | **ΔAIC_c_** | ***w*AIC_c_** | **Adj R^2^** |
| --- | --- | --- | --- | --- | --- | --- | --- | --- | --- | --- |
|  | **Independent variables** | |  |  |  |  |  |  |  |  |
| Path straightness | |  |  |  |  |  |  |  |  |  |
|  | RI |  |  |  |  | 3 | 463.88 | 0.00 | 0.57 | 0.03 |
|  | *Null* |  |  |  |  | 2 | 464.09 | 0.21 | 0.47 | 0 |
|  |  |  |  |  |  |  |  |  |  |  |
| Net displacement | |  |  |  |  |  |  |  |  |  |
|  | Tracking time + Temperature | | |  |  | 4 | 338.82 | 0 | 0.26 | 0.06 |
|  | Temperature | |  |  |  | 3 | 338.97 | 0.15 | 0.24 | 0.04 |
|  | Tracking time | |  |  |  | 3 | 338.98 | 0.16 | 0.24 | 0.04 |
|  | Sex + Rain | |  |  |  | 4 | 340.04 | 1.22 | 0.14 | 0.05 |
|  | *Null* |  |  |  |  | 2 | 340.16 | 1.35 | 0.13 | 0.00 |
|  |  |  |  |  |  |  |  |  |  |  |
| Total displacement | |  |  |  |  |  |  |  |  |  |
|  | **Tracking time** | |  |  |  | 3 | 337.12 | 0 | 0.98 | 0.11 |
|  | *Null* |  |  |  |  | 2 | 345.18 | 8.05 | 0.02 | 0 |
|  |  |  |  |  |  |  |  |  |  |  |

Table S5 - List of binomial GLMs explaining the probability of habitat selection in the Asian common toad in Toamasina (Madagascar). The table shows the number of parameters estimated in the model (K), AIC score of the model, AIC difference with the best model (ΔAIC), relative weight of the model with respect to the candidate set of models (*w*AIC), variation explained by the models adjusted for the number of parameters (Adj R^2^). Models including the quadratic distance of cell also include the linear term. The list of models was obtained excluding those models with a simpler version with lower AIC. Only models with ΔAIC < 2 and null models are shown. In bold, the significant variables.

| **Response** | |  | |  | |  | |  | | **K** | | **AIC** | | **ΔAIC** | | ***w*AIC** | | **Adj R^2^** | |  |
| --- | --- | --- | --- | --- | --- | --- | --- | --- | --- | --- | --- | --- | --- | --- | --- | --- | --- | --- | --- | --- |
|  | **Independent variables** | | |  | |  | |  | |  |  |  |  |  |  |  |  |  |  |  |
| Habitat characteristics | | |  | |  | |  | |  | |  | |  | |  | |  | |  | |
|  | **Distance cell^2^** | | |  | |  | |  | | 3 | | 522.245 | | 0.00 | | 1.00 | | 0.62 | |  |
|  | […] |  | |  | |  | |  | |  | |  | |  | |  | |  | |  |
|  | *Null* |  | |  | |  | |  | | 1 | | 1280.97 | | 758.72 | | 0.00 | | 0.00 | |  |
|  |  |  | |  | |  | |  | |  | |  | |  | |  | |  | |  |
| Habitat dissimilarities | |  | |  | |  | |  | |  | |  | |  | |  | |  | |  |
|  | **Distance cell^2^** + ΔBarren | | | | |  | |  | | 4 | | 521.19 | | 0.00 | | 0.63 | | 0.62 | |  |
|  | Distance cell^2^ | | |  | |  | |  | | 3 | | 522.24 | | 1.05 | | 0.37 | | 0.62 | |  |
|  | […] |  | |  | |  | |  | |  | |  | |  | |  | |  | |  |
|  | *Null* |  | |  | |  | |  | | 1 | | 1280.97 | | 759.77 | | 0.00 | | 0.00 | |  |

**References**

Moore M, Solofo Niaina Fidy JF, Edmonds D. 2015. The new toad in town: Distribution of the Asian toad, *Duttaphrynus melanostictus*, in the Toamasina area of eastern Madagascar. Tropical Conservation Science **8**:440–455.
